# 2,5-Bis(5-methyl­pyrazin-2-yl)-1,3,4-oxadiazole

**DOI:** 10.1107/S1600536811015029

**Published:** 2011-05-14

**Authors:** Yue Ju, Jing-Min Wu, Cheng-Peng Li, Jian-Hua Guo

**Affiliations:** a College of Chemistry, Tianjin Key Laboratory of Structure and Performance for Functional Molecule, Tianjin Normal University, Tianjin 300387, People’s Republic of China

## Abstract

In the title mol­ecule, C_12_H_10_N_6_O, the dihedral angle between the two pyrazine rings [planar to within 0.009 (3) and 0.018 (3) Å] is 5.62 (15)°. They deviate from the central oxadiazole ring [planar to within 0.005 (3) Å] by 1.52 (16) and 5.55 (17)°, respectively. In the crystal, C—H⋯N inter­actions involving the pyrazine rings connect mol­ecules to form zigzag supramolecular chains propagating in [010].

## Related literature

For background information and applications of oxadiazole derivatives, see: Schnurch *et al.* (2006 ▶); Crabtree (2005 ▶); Venkatakrishnan *et al.* (2000 ▶). For related oxadiazole derivatives, see: Du *et al.* (2005 ▶, 2006 ▶, 2009 ▶).
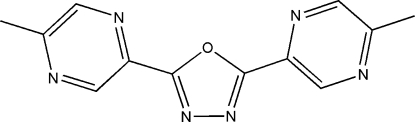


## Experimental

### 

#### Crystal data

C_12_H_10_N_6_O
*M* *_r_* = 254.26
Monoclinic, 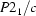

*a* = 3.9084 (8) Å
*b* = 19.054 (4) Å
*c* = 16.328 (4) Å
β = 101.64 (3)°
*V* = 1191.0 (5) Å^3^
*Z* = 4
Mo *K*α radiation
μ = 0.10 mm^−1^
*T* = 296 K
0.16 × 0.12 × 0.08 mm

#### Data collection

Bruker SMART CCD area-detector diffractometer
Absorption correction: multi-scan (*SADABS*; Sheldrick, 1996 ▶) *T*_min_ = 0.984, *T*_max_ = 0.992
6101 measured reflections
2108 independent reflections
1077 reflections with *I* > 2σ(*I*)
*R*_int_ = 0.074

#### Refinement

*R*[*F* ^2^ > 2σ(*F* ^2^)] = 0.064
*wR*(*F* ^2^) = 0.120
*S* = 1.00
2108 reflections
174 parameters
H-atom parameters constrained
Δρ_max_ = 0.15 e Å^−3^
Δρ_min_ = −0.18 e Å^−3^

### 

Data collection: *SMART* (Bruker, 2007 ▶); cell refinement: *SAINT* (Bruker, 2007 ▶); data reduction: *SAINT*; program(s) used to solve structure: *SHELXS97* (Sheldrick, 2008 ▶); program(s) used to refine structure: *SHELXL97* (Sheldrick, 2008 ▶); molecular graphics: *DIAMOND* (Brandenburg, 1999 ▶); software used to prepare material for publication: *SHELXTL* (Bruker, 2007 ▶).

## Supplementary Material

Crystal structure: contains datablocks I, global. DOI: 10.1107/S1600536811015029/su2270sup1.cif

Structure factors: contains datablocks I. DOI: 10.1107/S1600536811015029/su2270Isup2.hkl

Supplementary material file. DOI: 10.1107/S1600536811015029/su2270Isup3.cml

Additional supplementary materials:  crystallographic information; 3D view; checkCIF report

## Figures and Tables

**Table 1:** Hydrogen-bond geometry (Å, °)

*D*—H⋯*A*	*D*—H	H⋯*A*	*D*⋯*A*	*D*—H⋯*A*
C11—H11⋯N2^i^	0.93	2.59	3.414 (4)	148

Symmetry code: (i) 
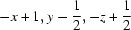
.

**Fig. 1.**
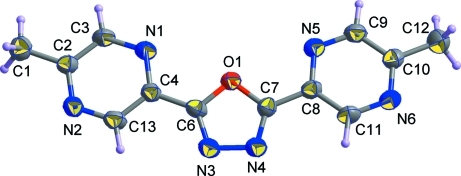
A view of the molecular structure of the title molecule with the numbering scheme. Displacement ellipsoids are drawn at the 50%probabilit level.

**Fig. 2.**
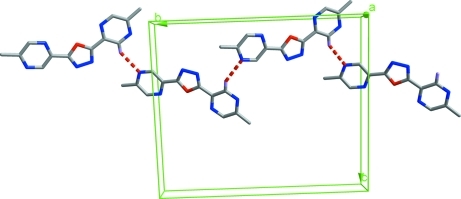
A partial view, along the a-axis, of the crystal packing in the title compound, showing the N-H···N hydrogen-bonds [red dashed lines; see Table 1 for details; H-atoms not involved in these interactions have been omitted for clarity].

## supplementary crystallographic information

### Comment

Currently derivatives of oxadiazole systems are of growing research interest, as they are precursors to functional N-heterocyclic compounds, as well as being used in pharmaceuticals as metabolically stable surrogates and photographically active systems (Schnurch *et al.*, 2006; Crabtree, 2005; Venkatakrishnan, *et al.*, 2000). Among them, oxadiazole compounds decorated by different groups on the 5-membered ring, such as pyridyl (Du, *et al.*, 2009) and pyrazinyl rings, (Du, *et al.*, 2005, 2006), also show interesting coordination behaviors. However, the related ligands involving methyl-pyrazinyl groups remain uninvestigated. They may display three typical configurations under different surroundings and multiple binding patterns (hexadentate at the most) during coordination. In this contribution, we present the crystal structure of the title compound, a bis(4-methylpyrazinyl) substituted oxadiazole. It was prepared by the reaction of 5-methylpyrazine-2-carboxylic acid and hydrazine dihydrochloride in the presence of polyphosphoric acid and anhydrous phosphorus pentoxide.

In the molecule of the title compound, Fig.1, pyrazinyl ring A [(N1,C3,C2,N2,C13,C4); planar to within 0.009 (3) Å] is inclined to pyrazinyl ring B [(N5,C9,C10,N6,C11,C9); planar to within 0.018 (3) Å] by 5.62 (15) °. They deviate from the central oxadiazole ring (planar to within 0.005 (3) Å) by 1.52 (16)° and 5.55 (17)°, respectively.

In the crystal a C—H···N interaction, involving pyrazinyl rings A and B, connect molecules to form zigzag poylmer chains propagating in [010] (Table 1 and Fig. 2).

### Experimental

5-Methylpyrazine-2-carboxylic acid (0.3 mol) and hydrazine dihydrochloride (0.2 mol) were mixed with stirring, to which polyphosphoric acid (85%, 60 ml) was added. Then, anhydrous phosphorus pentoxide (0.6 mol) was carefully added to the above mixture. The viscous solution was heated at 393 K, with stirring for *ca* 6 h. After cooling to room temperature, the resultant viscous liquid was poured over distilled water with stirring, dissolved, and then neutralized with sodium hydrate (3 mol/*L*). A large amount of orange precipitation of title compound was obtained and dried in air. Its single-crystal can be recrystallized from its methanol solution (Yield: 63%). Anal. Calc. for C_12_H_10_N_6_O: C, 55.69; H, 3.96; N, 33.05%. Found: C, 55.62; H, 4.01; N, 33.09%. Spectroscopic data for the title compound is given in the archived CIF.

### Refinement

All H atoms were initially located in a difference Fourier map. The C—H atoms were then constrained to an ideal geometry, and refind as riding atoms: C—H = 0.93 (CH_aromatic_) and 0.96 Å (CH_3_), with *U*iso(H) = 1.2*U*eq (C).

### Crystal data

**Table:**

C_12_H_10_N_6_O	*F*(000) = 528
*M**_r_* = 254.26	*D*_x_ = 1.418 Mg m^−^^3^
Monoclinic, *P*2_1_/*c*	Mo *K*α radiation, λ = 0.71073 Å
Hall symbol: -P 2ybc	Cell parameters from 460 reflections
*a* = 3.9084 (8) Å	θ = 2.3–22.4°
*b* = 19.054 (4) Å	µ = 0.10 mm^−^^1^
*c* = 16.328 (4) Å	*T* = 296 K
β = 101.64 (3)°	BLOCK, yellow
*V* = 1191.0 (5) Å^3^	0.16 × 0.12 × 0.08 mm
*Z* = 4	

### Data collection

**Table:**

Bruker SMART CCD area-detector diffractometer	2108 independent reflections
Radiation source: fine-focus sealed tube	1077 reflections with *I* > 2σ(*I*)
graphite	*R*_int_ = 0.074
φ and ω scans	θ_max_ = 25.0°, θ_min_ = 2.1°
Absorption correction: multi-scan (*SADABS*; Sheldrick, 1996)	*h* = −4→3
*T*_min_ = 0.984, *T*_max_ = 0.992	*k* = −22→22
6101 measured reflections	*l* = −13→19

### Refinement

**Table:**

Refinement on *F*^2^	Primary atom site location: structure-invariant direct methods
Least-squares matrix: full	Secondary atom site location: difference Fourier map
*R*[*F*^2^ > 2σ(*F*^2^)] = 0.064	Hydrogen site location: inferred from neighbouring sites
*wR*(*F*^2^) = 0.120	H-atom parameters constrained
*S* = 1.00	*w* = 1/[σ^2^(*F*_o_^2^) + (0.042*P*)^2^] where *P* = (*F*_o_^2^ + 2*F*_c_^2^)/3
2108 reflections	(Δ/σ)_max_ < 0.001
174 parameters	Δρ_max_ = 0.15 e Å^−^^3^
0 restraints	Δρ_min_ = −0.18 e Å^−^^3^

### Special details

**Table:**

Experimental. Spectroscopic data for the title compound: IR (KBr, cm^-1^): 3036m, 1515w, 1568w, 1461s, 1340w, 1272m, 1176m, 1092m, 1028?s, 915m, 835w, 769w, 728m, 522w.
Geometry. All e.s.d.'s (except the e.s.d. in the dihedral angle between two l.s. planes) are estimated using the full covariance matrix. The cell e.s.d.'s are taken into account individually in the estimation of e.s.d.'s in distances, angles and torsion angles; correlations between e.s.d.'s in cell parameters are only used when they are defined by crystal symmetry. An approximate (isotropic) treatment of cell e.s.d.'s is used for estimating e.s.d.'s involving l.s. planes.
Refinement. Refinement of *F*^2^ against ALL reflections. The weighted *R*-factor *wR* and goodness of fit *S* are based on *F*^2^, conventional *R*-factors *R* are based on *F*, with *F* set to zero for negative *F*^2^. The threshold expression of *F*^2^ > σ(*F*^2^) is used only for calculating *R*-factors(gt) *etc*. and is not relevant to the choice of reflections for refinement. *R*-factors based on *F*^2^ are statistically about twice as large as those based on *F*, and *R*- factors based on ALL data will be even larger.

### Fractional atomic coordinates and isotropic or equivalent isotropic displacement parameters (Å^2^)

**Table:**

	*x*	*y*	*z*	*U*_iso_*/*U*_eq_	
O1	0.3651 (5)	0.85150 (10)	0.39441 (13)	0.0419 (6)	
N1	0.1455 (7)	0.98781 (14)	0.40516 (16)	0.0502 (8)	
N2	0.1510 (7)	1.08251 (13)	0.27453 (17)	0.0457 (7)	
N3	0.4865 (7)	0.86734 (14)	0.26968 (17)	0.0513 (8)	
N4	0.5726 (7)	0.79743 (14)	0.29485 (17)	0.0511 (8)	
N5	0.4644 (7)	0.73687 (13)	0.49788 (18)	0.0471 (8)	
N6	0.7282 (8)	0.61027 (13)	0.44708 (18)	0.0503 (8)	
C1	−0.0765 (8)	1.17661 (16)	0.3485 (2)	0.0551 (10)	
H1A	0.0961	1.2080	0.3350	0.083*	
H1B	−0.1100	1.1863	0.4041	0.083*	
H1C	−0.2932	1.1831	0.3094	0.083*	
C2	0.0444 (8)	1.10226 (16)	0.3440 (2)	0.0387 (8)	
C3	0.0457 (8)	1.05456 (17)	0.4080 (2)	0.0495 (9)	
H3	−0.0274	1.0699	0.4557	0.059*	
C4	0.2502 (8)	0.96885 (15)	0.3355 (2)	0.0386 (8)	
C6	0.3694 (8)	0.89630 (17)	0.3300 (2)	0.0411 (9)	
C7	0.4940 (8)	0.79085 (16)	0.3676 (2)	0.0403 (8)	
C8	0.5402 (8)	0.72924 (16)	0.4219 (2)	0.0398 (8)	
C9	0.5263 (8)	0.68055 (17)	0.5472 (2)	0.0485 (9)	
H9	0.4742	0.6833	0.6002	0.058*	
C10	0.6641 (8)	0.61806 (17)	0.5240 (2)	0.0435 (9)	
C11	0.6639 (8)	0.66627 (17)	0.3962 (2)	0.0485 (9)	
H11	0.7035	0.6627	0.3421	0.058*	
C12	0.7464 (9)	0.55814 (17)	0.5838 (2)	0.0644 (11)	
H12A	0.5515	0.5265	0.5760	0.097*	
H12B	0.7918	0.5757	0.6401	0.097*	
H12C	0.9489	0.5338	0.5737	0.097*	
C13	0.2561 (8)	1.01613 (17)	0.2717 (2)	0.0465 (9)	
H13	0.3368	1.0011	0.2248	0.056*	

### Atomic displacement parameters (Å^2^)

**Table:**

	*U*^11^	*U*^22^	*U*^33^	*U*^12^	*U*^13^	*U*^23^
O1	0.0517 (15)	0.0398 (14)	0.0370 (14)	−0.0001 (10)	0.0157 (11)	−0.0021 (11)
N1	0.072 (2)	0.0464 (18)	0.0363 (18)	0.0037 (15)	0.0208 (17)	0.0015 (14)
N2	0.058 (2)	0.0384 (17)	0.0435 (19)	−0.0050 (14)	0.0157 (16)	−0.0006 (14)
N3	0.072 (2)	0.0440 (18)	0.043 (2)	−0.0048 (14)	0.0242 (17)	−0.0012 (15)
N4	0.071 (2)	0.0427 (18)	0.046 (2)	−0.0013 (15)	0.0256 (17)	−0.0026 (15)
N5	0.059 (2)	0.0433 (18)	0.0426 (18)	0.0011 (14)	0.0198 (16)	−0.0029 (15)
N6	0.062 (2)	0.0465 (18)	0.045 (2)	0.0080 (15)	0.0184 (16)	−0.0027 (16)
C1	0.058 (3)	0.047 (2)	0.061 (3)	−0.0026 (17)	0.013 (2)	−0.0034 (19)
C2	0.040 (2)	0.041 (2)	0.035 (2)	−0.0037 (16)	0.0081 (17)	−0.0045 (17)
C3	0.065 (2)	0.050 (2)	0.038 (2)	0.003 (2)	0.022 (2)	−0.0031 (19)
C4	0.043 (2)	0.037 (2)	0.037 (2)	−0.0047 (15)	0.0114 (18)	−0.0020 (16)
C6	0.047 (2)	0.043 (2)	0.035 (2)	−0.0105 (17)	0.0138 (18)	−0.0013 (17)
C7	0.047 (2)	0.033 (2)	0.041 (2)	−0.0037 (16)	0.0119 (18)	−0.0064 (18)
C8	0.044 (2)	0.038 (2)	0.039 (2)	−0.0026 (16)	0.0116 (17)	−0.0086 (17)
C9	0.061 (2)	0.050 (2)	0.039 (2)	−0.0018 (18)	0.0210 (19)	−0.0048 (19)
C10	0.041 (2)	0.047 (2)	0.044 (2)	−0.0004 (17)	0.0124 (19)	−0.0026 (18)
C11	0.060 (2)	0.051 (2)	0.038 (2)	0.0036 (18)	0.0179 (19)	−0.0081 (18)
C12	0.073 (3)	0.064 (3)	0.058 (3)	0.014 (2)	0.019 (2)	0.011 (2)
C13	0.060 (2)	0.049 (2)	0.035 (2)	−0.0086 (18)	0.0175 (19)	−0.0057 (18)

### Geometric parameters (Å, °)

**Table:**

O1—C6	1.357 (3)	C1—H1C	0.9600
O1—C7	1.367 (3)	C2—C3	1.384 (4)
N1—C3	1.334 (4)	C3—H3	0.9300
N1—C4	1.334 (4)	C4—C13	1.381 (4)
N2—C13	1.334 (4)	C4—C6	1.467 (4)
N2—C2	1.339 (4)	C7—C8	1.460 (4)
N3—C6	1.291 (4)	C8—C11	1.390 (4)
N3—N4	1.415 (3)	C9—C10	1.390 (4)
N4—C7	1.293 (4)	C9—H9	0.9300
N5—C9	1.334 (4)	C10—C12	1.494 (4)
N5—C8	1.340 (4)	C11—H11	0.9300
N6—C10	1.338 (4)	C12—H12A	0.9600
N6—C11	1.344 (4)	C12—H12B	0.9600
C1—C2	1.500 (4)	C12—H12C	0.9600
C1—H1A	0.9600	C13—H13	0.9300
C1—H1B	0.9600		
			
C6—O1—C7	102.8 (2)	N4—C7—O1	112.6 (3)
C3—N1—C4	115.4 (3)	N4—C7—C8	127.8 (3)
C13—N2—C2	116.6 (3)	O1—C7—C8	119.6 (3)
C6—N3—N4	106.3 (2)	N5—C8—C11	121.9 (3)
C7—N4—N3	105.8 (3)	N5—C8—C7	116.8 (3)
C9—N5—C8	115.1 (3)	C11—C8—C7	121.3 (3)
C10—N6—C11	116.4 (3)	N5—C9—C10	123.9 (3)
C2—C1—H1A	109.5	N5—C9—H9	118.1
C2—C1—H1B	109.5	C10—C9—H9	118.1
H1A—C1—H1B	109.5	N6—C10—C9	120.4 (3)
C2—C1—H1C	109.5	N6—C10—C12	118.2 (3)
H1A—C1—H1C	109.5	C9—C10—C12	121.4 (3)
H1B—C1—H1C	109.5	N6—C11—C8	122.1 (3)
N2—C2—C3	120.0 (3)	N6—C11—H11	118.9
N2—C2—C1	117.5 (3)	C8—C11—H11	118.9
C3—C2—C1	122.4 (3)	C10—C12—H12A	109.5
N1—C3—C2	123.8 (3)	C10—C12—H12B	109.5
N1—C3—H3	118.1	H12A—C12—H12B	109.5
C2—C3—H3	118.1	C10—C12—H12C	109.5
N1—C4—C13	121.4 (3)	H12A—C12—H12C	109.5
N1—C4—C6	117.6 (3)	H12B—C12—H12C	109.5
C13—C4—C6	121.0 (3)	N2—C13—C4	122.7 (3)
N3—C6—O1	112.6 (3)	N2—C13—H13	118.7
N3—C6—C4	127.9 (3)	C4—C13—H13	118.7
O1—C6—C4	119.5 (3)		
			
C6—N3—N4—C7	0.9 (4)	C6—O1—C7—C8	178.2 (3)
C13—N2—C2—C3	−0.3 (5)	C9—N5—C8—C11	1.9 (5)
C13—N2—C2—C1	−179.7 (3)	C9—N5—C8—C7	−176.8 (3)
C4—N1—C3—C2	0.5 (5)	N4—C7—C8—N5	174.8 (3)
N2—C2—C3—N1	−0.7 (5)	O1—C7—C8—N5	−2.6 (4)
C1—C2—C3—N1	178.7 (3)	N4—C7—C8—C11	−4.0 (5)
C3—N1—C4—C13	0.6 (5)	O1—C7—C8—C11	178.7 (3)
C3—N1—C4—C6	178.8 (3)	C8—N5—C9—C10	0.9 (5)
N4—N3—C6—O1	−0.6 (4)	C11—N6—C10—C9	2.3 (5)
N4—N3—C6—C4	178.9 (3)	C11—N6—C10—C12	−177.4 (3)
C7—O1—C6—N3	0.1 (4)	N5—C9—C10—N6	−3.2 (5)
C7—O1—C6—C4	−179.5 (3)	N5—C9—C10—C12	176.5 (3)
N1—C4—C6—N3	−178.7 (3)	C10—N6—C11—C8	0.5 (5)
C13—C4—C6—N3	−0.5 (5)	N5—C8—C11—N6	−2.8 (5)
N1—C4—C6—O1	0.8 (4)	C7—C8—C11—N6	175.9 (3)
C13—C4—C6—O1	179.0 (3)	C2—N2—C13—C4	1.4 (5)
N3—N4—C7—O1	−0.9 (4)	N1—C4—C13—N2	−1.7 (5)
N3—N4—C7—C8	−178.4 (3)	C6—C4—C13—N2	−179.8 (3)
C6—O1—C7—N4	0.5 (4)		

### Hydrogen-bond geometry (Å, °)

**Table:**

*D*—H···*A*	*D*—H	H···*A*	*D*···*A*	*D*—H···*A*
C11—H11···N2^i^	0.93	2.59	3.414 (4)	148

Symmetry codes: (i) −*x*+1, *y*−1/2, −*z*+1/2.
